# Linguistic Isolation and Mortality in Older Mexican Americans: Findings from the Hispanic Established Populations Epidemiologic Studies of the Elderly

**DOI:** 10.1089/heq.2020.0139

**Published:** 2021-06-01

**Authors:** Donglan Zhang, Janani Rajbhandari-Thapa, Saswat Panda, Zhuo Chen, Lu Shi, Yan Li, Ye Shen, Ramesh Ghimire, Kerstin Gerst Emerson

**Affiliations:** ^1^ Department of Health Policy and Management, College of Public Health, University of Georgia, Athens, Georgia, USA.; ^2^ Department of Public Health Sciences, Clemson University, Clemson, South Carolina, USA.; ^3^ Department of Population Health Science and Policy, Gynecology, and Reproductive Science (Secondary), Icahn School of Medicine at Mount Sinai, New York, New York, USA.; ^4^ Department of Obstetrics, Gynecology, and Reproductive Science (Secondary), Icahn School of Medicine at Mount Sinai, New York, New York, USA.; ^5^ Department of Epidemiology and Biostatistics, College of Public Health, University of Georgia, Athens, Georgia, USA.; ^6^ Atlanta Regional Commission, Atlanta, Georgia, USA.

**Keywords:** hazards survival analysis, Mexican Americans, linguistic isolation

## Abstract

**Purpose:** Limited English proficiency and increased language isolation are known to be associated with adverse health outcomes. It is not clear how neighborhood-level linguistic isolation may impact individual health and risk of death among Hispanic older adults. We examined the link between living in a linguistically isolated neighborhood and all-cause mortality among an older Mexican American cohort.

**Methods:** Using a longitudinal sample of older Mexican Americans from the Hispanic Established Populations for the Epidemiologic Studies of the Elderly, we calculated the days from the baseline interview (1993–1994) until observed death through five waves of follow-up (until 2004–2005) using Cox regression. A linguistically isolated neighborhood was defined as a census tract with more than 30% of linguistically isolated households.

**Results:** Our results showed that living in a neighborhood with more than 30% of linguistically isolated households predicted higher mortality (hazard ratio: 1.25; 95% confidence interval: 1.04–1.50), after adjusting for age, sex, nativity, years of education, marital status, self-reported health status, number of chronic conditions, ever smoked, ever drank, and other neighborhood-level contextual factors.

**Conclusion:** Living in a neighborhood with a high proportion of linguistically isolated households predicted higher mortality among older Mexican Americans. Addressing the social capital shortage in linguistically isolated neighborhoods is one way to address health disparities in the United States.

## Introduction

Inadequate or poor English language proficiency is one of the social determinants of health in the United States that hinders residents from establishing and maintaining positive health outcomes and leads to health inequities.^1^ It has been documented that limited-English proficient older adults had significantly worse outcomes in health status and access to health care than older adults who speak English only.^2^

While there are a number of definitions, linguistic isolation can be divided into three main components: (a) limited English proficiency, (b) solitary living, and (c) limited English of other adult household members.^3^ It is known that limited English proficiency and increased language isolation are associated with adverse health outcomes.^4^ For example, language isolation is found to be associated with an increased risk of depressive symptoms.^5^ Language barrier is also linked with an increased risk of delaying care, and forgoing preventive services such as pap tests, and thus resulting in overall worse health status.^2,6^

Older Latinos may be particularly vulnerable to linguistic isolation. More than half of the Latinos older than 65 years of age in the United States do not speak English proficiently,^7^ and Mexican Americans have the lowest level of English proficiency among major Latino subgroups.^8^ Few studies have examined the link between linguistic isolation and health and mortality among Hispanic older adults. It has been reported that older Latinos suffer from a higher prevalence of depression and other mental health disorders, but they are largely underdiagnosed and underserved, partly due to their lack of English language proficiency coupled with a lack of culturally competent and bilingual clinicians.^9,10^ For example, individuals who cannot speak English well often have trouble communicating with health service providers. Linguistically isolated individuals might not receive needed health care services and information on time.^11,12^ Therefore, it is important to study the adverse impact of linguistic isolation on their health.

Furthermore, most prior literature so far measured English proficiency or linguistic isolation at the individual or the household level. With the increase of Latino-white segregation in certain parts of the United States,^13^ it is not clear how neighborhood-level percent of linguistically isolated households may impact individual health and risk of death. Linguistic isolation, compounded with limited access to health care, may be making the Latino communities disproportionately vulnerable to diseases and associated deaths.

In the present study, we hypothesized that older Mexican Americans living in a community with a high percentage of linguistically isolated residents have worse health conditions and thus have higher all-cause mortality than those living in a less linguistically isolated environment. We empirically test this premise using data from the Hispanic Established Populations for the Epidemiologic Studies of the Elderly (H-EPESE) survey.

## Methods

### Data

H-EPESE is a publically available dataset containing only deidentified information. The baseline survey was conducted in 1993–1994, on a representative sample of community-dwelling Mexican Americans aged 65 years and older, residing in five southwestern states (Arizona, California, Colorado, New Mexico, and Texas). We used the original baseline sample with respondents followed-up for five waves (through 2004–2005). Because 70.71% (816 out of 1154) of participants died in the fifth wave, we did not use the recent 2012–2013 wave of H-EPSE when the majority of our study population deceased. This is the largest longitudinal dataset of older Mexican Americans available containing neighborhood level data. The study was reviewed and approved by the University of Texas Medical Branch Institutional Review Board.

We calculated the mortality rate and the time-to-event variable as the days from the baseline interview until the respondent died or was censored. We defined being censored as a respondent being alive during the fifth interview. The predictor of interest was living in a census tract with more than 30% of linguistically isolated households. We used the U.S. Census Bureau definition of linguistically isolated household, which defines it as any household with either (a) no person aged 14 and older speaking only English at home or (b) among those who speak another language at home, no person aged 14 and older speaks English very well.^14^ We used the cutoff point of 30% because the U.S. Census Bureau defined those neighborhoods as tracts with the highest level of linguistic isolation.^14^

### Data analysis

We performed survival analysis using Cox regression models to assess whether linguistic isolation affects mortality. We adjusted for age, sex, nativity, years of education, marital status, self-reported health status, number of chronic conditions (e.g., hypertension, diabetes, stroke, heart failure, heart attack, arthritis, cancer, and hip fracture), ever smoked, ever drank, percent of immigrants in the neighborhood, neighborhood poverty rate, and percent of Mexican Americans in the neighborhood. The neighborhood-level contextual factors were selected based on the available measures in this dataset. Since the baseline sample in the 1993–1994 wave did not collect this information, we did not distinguish the individuals who spoke mostly Spanish or English in the target population of analysis. Individual sampling weights calculated at baseline were used to estimate the distribution of individual and neighborhood characteristics. Since the neighborhood-level contextual factors were measured at the baseline wave in 1993–1994, we would like to assess whether moving to a different neighborhood would affect the studied association. Among the study sample, around 14.1% (*N*=163) reported having moved since the last interview. As a sensitivity analysis, we included this variable—whether they have moved—in the regression to assess the robustness of the findings. Data analyses took place in 2019, and we used Stata 14.2 (StataCorp, College Station, TX) for all the analyses.

## Results

The complete study sample consisted of 1154 Mexican Americans aged 65 and older. Around 17.6% of the respondents lived in a linguistically isolated neighborhood, adjusting for baseline sampling weights. A marginally significant hazard ratio (HR) was observed for living in a neighborhood with more than 30% of linguistically isolated households predicting a higher risk of death (HR=1.14, 95% confidence interval [CI]: 0.98–1.32; Table 1). About 58.8% of the respondents lived in a neighborhood that is composed of more than 30% of immigrants, compared to 41.2% of the study population who lived in a neighborhood with fewer than 30% of immigrant residents, 31.3% lived in a high-poverty neighborhood (≥30% of the residents living in poverty), and 45.8% lived in a neighborhood with no <60% residents being Mexican Americans. The respondents aged 70.2 years on average, with about 59.4% of the respondents being women, 55.6% U.S.-born, 19.0% high school graduates, 45.2% being married, and about 14.1% who had moved. About 65.6% of the respondents reported fair or poor health, 39.4% reported more than two chronic conditions, 48.1% of them had smoked at least 100 cigarettes in their entire life, and 62.6% had ever drunk any type of alcohol.

**Table 1.** Survival-Related Neighborhood and Individual Characteristics and Results from Univariate Hazards Survival Analysis in the Cohort of Older Mexican Americans, Hispanic Established Populations for the Epidemiologic Studies of the Elderly (*N*=1154)

	Sample distribution^a^		Univariate survival analyses	
	N/mean^b^	%/SE^b^	HR	95% CI
Neighborhood characteristics				
Linguistic isolated households				
<30%	951	82.4	1.00	
≥30%	203	17.6	1.14	0.98–1.32
Immigrant population				
<30%	475	41.2	1.00	
≥30%	679	58.8	0.97	0.85–1.11
Poverty rate				
<30%	793	68.7	1.00	
≥30%	361	31.3	0.99	0.87–1.14
Mexican Americans				
<60%	625	54.2	1.00	
≥60%	529	45.8	1.00	0.85–1.18
Individual characteristics				
Age (years)	70.2	0.2	1.05	1.04–1.07
Gender				
Male	469	40.6	1.00	
Female	685	59.4	0.87	0.75–1.00
Nativity				
Mexico-born	512	44.4	1.00	
U.S.-born	642	55.6	0.96	0.83–1.10
Education level				
Less than high school	935	81.0	1.00	
High school graduates	219	19.0	1.01	0.79–1.30
Marital status				
Not married/widowed/separated	632	54.8	1.00	
Married	522	45.2	0.99	0.86–1.14
Have moved since the last interview				
No	991	85.9	1.00	
Yes	163	14.1	0.89	0.73–1.08
Health conditions				
Self-rated health				
Excellent	77	6.7	1.00	
Good	319	27.6	1.07	0.79–1.45
Fair	584	50.6	1.21	0.91–1.61
Poor	174	15.1	1.68	1.23–2.28
Number of chronic conditions^c^				
0	99	8.6	1.00	
1–2	600	52.0	1.11	0.87–1.41
>2	455	39.4	1.43	1.12–1.82
Have smoked at least 100 cigarettes				
No	599	51.9	1.00	
Yes	555	48.1	1.05	0.92–1.21
Have drunk any type of alcohol				
No	432	37.4	1.00	
Yes	722	62.6	0.96	0.84–1.10

^a^ Baseline sampling weights were used in calculating sample distribution.

^b^ Age distribution was presented as mean and SE.

^c^ Chronic conditions included hypertension, diabetes, stroke, hip fracture, arthritis, cancer, heart attack, and heart failure.

CI, confidence interval; HR, hazard ratio; SE, standard error.

Living in a neighborhood with more than 30% of linguistically isolated households predicted significantly higher mortality with an adjusted HR of 1.24 (95% CI: 1.04–1.48, *p*=0.017) after adjusting for other neighborhood characteristics (Table 2). After further adjusting for individual characteristics, the HR was 1.25 (95% CI: 1.04–1.50, *p*=0.016). Taking into consideration of a respondent's relocation during the study period did not change the findings, in other words, the baseline exposure to neighborhood contextual factors still had an impact on health and life expectancy among older adults. Age was significantly associated with a higher HR (1.05, 95% CI: 1.04–1.07), while being female was associated with a lower HR (0.77, 95% CI: 0.64–0.93). Self-reported poor health was associated with a higher HR (1.41, 95% CI: 1.02–1.97), and having more than two chronic conditions was associated with a significantly higher HR (1.52, 95% CI: 1.17–1.98). Other individual and neighborhood predictors were not significant in the regression.

**Table 2.** Neighborhoods with High Rates of Linguistic Isolation and Mortality in the Cohort of Older Mexican Americans: Results from Multivariate Hazards Survival Analysis, Hispanic Established Populations for the Epidemiologic Studies of the Elderly (*N*=1154)

	Model 1^a^		Model 2^b^	
	HR	95% CI	HR	95% CI
Neighborhood characteristics				
Linguistic isolated households				
<30%	1.00		1.00	
≥30%	1.24	1.04–1.48	1.25	1.04–1.50
Immigrant population				
<30%	1.00		1.00	
≥30%	0.91	0.79–1.06	0.92	0.79–1.07
Poverty rate				
<30%	1.00		1.00	
≥30%	0.93	0.93–1.09	0.88	0.75–1.04
Mexican Americans				
<60%	1.00		1.00	
≥60%	0.97	0.81–1.16	1.02	0.84–1.23
Individual characteristics				
Age (years)			1.05	1.04–1.07
Gender				
Male			1.00	
Female			0.77	0.64–0.93
Nativity				
Mexico-born				
U.S.-born			1.04	0.90–1.21
Education level				
Less than high school				
High school graduates			1.12	0.87–1.44
Marital status				
Not married/widowed/separated				
Married			0.99	0.84–1.16
Have moved since the last interview				
Yes			1.00	
No			0.92	0.75–1.12
Health conditions				
Self-rated health				
Excellent			1.00	
Good			1.01	0.74–1.38
Fair			1.14	0.84–1.54
Poor			1.41	1.02–1.97
Number of chronic conditions^c^				
0			1.00	
1–2			1.19	0.92–1.53
>2			1.52	1.17–1.98
Have smoked at least 100 cigarettes				
No			1.00	
Yes			1.02	0.87–1.20
Have drunk any type of alcohol				
No			1.00	
Yes			0.90	0.76–1.07

^a^ Hazards survival analysis in Model 1 adjusted for neighborhood characteristics.

^b^ Hazards survival analysis in Model 2 adjusted for both neighborhood and individual characteristics.

^c^ Chronic conditions included hypertension, diabetes, stroke, hip fracture, arthritis, cancer, heart attack, and heart failure.

## Discussion

We found that living in a neighborhood with a high proportion of linguistically isolated households was significantly associated with higher mortality among older Mexican Americans after controlling for both individual and neighborhood contextual variables. This finding, while novel in establishing the association between linguistic isolation and mortality among older Mexican Americans, was consistent with previous studies that identified associations of linguistic isolation with poor mental health,^5^ and with poor access to health services such as access to the active kidney transplant waiting list.^15^ A qualitative study reported that older Latinos often identified inadequate language proficiency as a major life stressor, which could lead to depression.^10^

It is worth noting, however, that the relationship between linguistic isolation and health outcomes is complex and vary based on context. For instance, a longitudinal study in the United States found that children in English-dominant homes did not necessarily have better developmental outcomes than children in non-English dominant households.^4^ Under certain circumstances, linguistic isolation at the community-level might provide social and cultural capital for the community residents to have a cultural buffer zone, where they could more easily adjust to the broader United States context: a multilevel study of Los Angeles County residents found that foreign-born Latinos (who spent 15 years or more in the United States) and U.S.-born Latinos residing in census tracts with high linguistic isolation were less likely to have depression.^16^

One possible explanation for our finding is that older adults living in linguistically isolated households may be socially isolated and lonely.^3,14,17,18^ Research has consistently found that loneliness is a risk factor for premature mortality.^19–22^ In addition, those living in linguistically isolated communities may have lower access to social capital, making it difficult for people to receive informal social support and maintain healthy lifestyles.^23,24^ This was further confirmed by a previous study that showed that the concentration of linguistically isolated households in a neighborhood was negatively associated with social capital.^23^ It is also possible that older adults living in linguistically isolated households are more likely to live in ethnic enclaves with neighborhood poverty, which would lead to worse health outcomes.^25^ Finally, living in linguistically isolated households means more language barriers to health care access, and the resulting negative consequences are worse among older adults.^26^

### Limitations

Several limitations should be considered when interpreting the results of the present study. First, the most recent data points in the analyses were from 2004/5 and may therefore not capture the current status of older Mexican Americans. However, the dataset allows us to answer a relevant and novel research question using the most recent data available for this purpose. There is a dearth of multilevel longitudinal data on older Mexican Americans, and the H-EPESE data are unique in allowing us to establish an important connection between linguistic isolation and mortality in this vulnerable population. Therefore, while the exact proportions may not precisely capture the current state of Mexican Americans, the dataset allowed us to determine a significant relationship between linguistic isolation and mortality. This is a notable contribution to the literature. Second, given the observational nature of the dataset, we cannot claim that the result was a causal relationship. Future studies need to use a causal approach such as instrumental variables approach or regression discontinuity approach to control for potential selection bias. Third, the H-EPESE sample was recruited from the four U.S. states that had the largest percentage of Mexican Americans, including Texas, California, New Mexico, Arizona, and also from Colorado, separately.^27^ Therefore, the study findings may not be generalizable to the entire Mexican American population in the United States. Nevertheless, the H-EPESE sample might still be the most suitable dataset to test this hypothesis because other states may not contain an adequate number of neighborhoods with more than 30% linguistically isolated households. Finally, we posit that older Mexican Americans living in linguistically isolated households are socially isolated and have lower social capital, which can decrease knowledge/access to services and social support, resulting in higher mortality. However, certain variables were not available in this dataset, and thus we were not able to explore the mechanisms such as social capital, access to health care, loneliness, and mental health that may affect health and longevity. In addition, behaviors and lifestyles are important predictors of longevity^28,29^; however, smoking and drinking alcohol were not measured in great detail and so only serve as limited control variables. Future research should, therefore, directly explore the pathways between social capital, loneliness, lifestyles, and mortality, as well as examining the impact of viable interventions to alleviate the burden of language barriers and linguistic isolation. In addition, census tracts and neighborhoods may be different levels of geographic aggregation. Further studies may explore measures at multiple geographic-aggregated levels.^30^

### Recommendations for improving practice

Establishing the importance of neighborhood context on health and mortality has important public health implications. Our finding suggests that older Mexican Americans living in linguistically isolated households are particularly vulnerable. At a time of limited resources, intervention targeted at these communities should focus not only on sharing availability of resources but also on linguistic services to access available resources. Access to language assistance for persons with limited English proficiency is required according to the Title VI of the Civil Rights Act and Executive Order 13166 issued in 2000. Any programs and agencies that receive federal support are required to provide language assistance to anyone who might have language barriers to access their services and products.^31^ In the medical care setting, for instance, health-related information and health care services (such as instructions to take medications and home visits by health care professionals) should be presented in languages understandable by older linguistically isolated adults. For some areas, interpreters and translation services are commonly available, while in other places, it is challenging to access interpretation services.^32,33^ Language barriers to health care access may be worse for linguistically isolated households.

Community organizations will need to think creatively about how to access immigrant-friendly services, such as working with universities and hospitals to deliver immigrant-made educational materials^34^ and using telephone interpreter services.^35^ Meanwhile, health care providers need to consider multilingual delivery of telemedicine services,^36^ especially when many health care services have been switched to the mode of telehealth delivery.^37^ Typically, third-party payers do not reimburse for interpretation services, and many organizations have to end up funding their own services.^38^ Therefore, stakeholders of senior health (hospitals/clinics, support agencies, government, etc.) should consider language assistance to older adults as a high-priority intervention, whereas payers should also consider reimbursing for interpretation services because such services have been shown to improve health care quality and clinical outcomes.^39^

While it is established that linguistic integration has a positive long-term effect on health outcomes, there was a gap in the literature examining the link between linguistic isolation and mortality among older Mexican adults. The findings of this study show higher mortality among older Mexican Americans living in a neighborhood with a high proportion of linguistically isolated households. Addressing the shortage of language and translation services in linguistically isolated neighborhoods may be one way to reducing health inequities in the United States, which is one of the overarching goals of Healthy People 2030.^40^ Old Mexican Americans are a particularly vulnerable group of older adults since they are more likely to be socioeconomically disadvantaged and more likely to have chronic health conditions compared to non-Hispanic whites. Future studies need to further examine the possible causal link between living in areas with a high level of linguistic isolation and higher mortality and identify interventions to reduce linguistic isolation or its behavioral ramifications.

## Abbreviations Used

CI: confidence interval
H-EPESE: Hispanic Established Populations for the Epidemiologic Studies of the Elderly
HR: hazard ratio
SE: standard error

## References

[1] Maleku A, Kim YK, Lee G. Social cohesion and immigrant health: does language-efficacy matter? Int J Migr Health So. 2019;15:17–30

[2] Ponce NA, Hays RD, Cunningham WE. Linguistic disparities in health care access and health status among older adults. J Gen Intern Med. 2006;21:786–791 16808783 10.1111/j.1525-1497.2006.00491.x PMC1924691

[3] Gubernskaya Z, Treas J. Pathways to linguistic isolation among older U.S. immigrants: assessing the role of living arrangements and English proficiency. J Gerontol B Psychol Sci Soc Sci. 2020;75:351–356 29373728 10.1093/geronb/gbx178

[4] Glick JE, Walker L, Luz L. Linguistic isolation in the home and community: Protection or risk for young children? Soc Sci Res. 2013;42:140–154 23146603 10.1016/j.ssresearch.2012.08.003 PMC3499731

[5] Ward JB, Albrecht SS, Robinson WR, et al. Neighborhood language isolation and depressive symptoms among elderly US Latinos. Ann Epidemiol. 2018;28:774–782 30201290 10.1016/j.annepidem.2018.08.009 PMC6215715

[6] Ponce NA, Chawla N, Babey SH, et al. Is there a language divide in pap test use? Med Care. 2006;44:998–1004 17063131 10.1097/01.mlr.0000233676.61237.ef

[7] Krogstad JM, Stepler R, Lopez MH. English proficiency on the rise among latinos. Pew Research Center Hispanic Trends, 2016. Available at http://www.pewhispanic.org/2015/05/12/english-proficiency-on-the-rise-among-latinos/ (Accessed 121, 2021)

[8] Tran VC. English Gain vs. Spanish Loss? Language assimilation among second-generation Latinos in young adulthood. Soc Forces. 2010;89:257–284

[9] Alvarez P, Rengifo J, Emrani T, et al. Latino older adults and mental health: a review and commentary. Clin Gerontol. 2014;37:33–48

[10] Sadule-Rios N, Tappen R, Williams CL, et al. Older Hispanics' explanatory model of depression. Arch Psychiatr Nurs. 2014;28:242–249 25017557 10.1016/j.apnu.2014.03.006

[11] Escarce JJ, Kapur K. Access to and quality of health care. In: Hispanics and the Future of America. Edited by Tienda M, Mitchell F. Washington, DC: National Academies Press, 2006, pp. 410–446 20669436

[12] González HM, Haan MN, Hinton L. Acculturation and the prevalence of depression in older Mexican Americans: baseline results of the Sacramento Area Latino Study on Aging. J Am Geriatr Soc. 2001;49:948–953 11527487 10.1046/j.1532-5415.2001.49186.x

[13] Anacker K, Niedt C, Kwon C. Analyzing segregation in mature and developing suburbs in the United States. J Urban Affairs. 2017;39:819–832

[14] Siegel P, Martin E, Bruno R. Language Use and Linguistic Isolation: Historical Data and Methodological Issues. Washington, DC: U.S. Census Bureau, 2001

[15] Talamantes E, Norris KC, Mangione CM, et al. Linguistic isolation and access to the active kidney transplant waiting list in the United States. Clin J Am Soc Nephrol. 2017;12:483–492 28183854 10.2215/CJN.07150716 PMC5338711

[16] Vega WA, Ang A, Rodriguez MA, et al. Neighborhood protective effects on depression in Latinos. Am J Commun Psychol. 2011;47:114–126 10.1007/s10464-010-9370-5 21052825

[17] Hodgson S, Watts I, Fraser S, et al. Loneliness, social isolation, cardiovascular disease and mortality: a synthesis of the literature and conceptual framework. J R Soc Med. 2020;113:185–192 32407646 10.1177/0141076820918236 PMC7366328

[18] Link MW, Mokdad AH, Stackhouse HF, et al. PEER REVIEWED: race, ethnicity, and linguistic isolation as determinants of participation in public health surveillance surveys. Prevent Chron Dis. 2006;3:A09 PMC1500943 16356362

[19] Luo Y, Waite LJ. Loneliness and mortality among older adults in China. J Gerontol Ser B Psychol Sci Soc Sci. 2014;69:633–645 24550354 10.1093/geronb/gbu007 PMC4049147

[20] Tanskanen J, Anttila T. A prospective study of social isolation, loneliness, and mortality in Finland. Am J Public Health. 2016;106:2042–2048 27631736 10.2105/AJPH.2016.303431 PMC5055788

[21] Beller J, Wagner A. Loneliness, social isolation, their synergistic interaction, and mortality. Health Psychol. 2018;37:808 30138019 10.1037/hea0000605

[22] Steptoe A, Shankar A, Demakakos P, et al. Social isolation, loneliness, and all-cause mortality in older men and women. Proc Natl Acad Sci USA. 2013;110:5797–5801 23530191 10.1073/pnas.1219686110 PMC3625264

[23] Nawyn SJ, Gjokaj L, Agbenyiga DL, et al. Linguistic isolation, social capital, and immigrant belonging. J Contemp Ethnogr. 2012;41:255–282

[24] Sundquist K, Hamano T, Li X, et al. Linking social capital and mortality in the elderly: a Swedish national cohort study. Exp Gerontol. 2014;55:29–36 24632181 10.1016/j.exger.2014.03.007

[25] Osypuk TL, Bates LM, Acevedo-Garcia D. Another Mexican birthweight paradox? The role of residential enclaves and neighborhood poverty in the birthweight of Mexican-origin infants. Soc Sci Med. 2010;70:550–560 19926186 10.1016/j.socscimed.2009.10.034 PMC2815074

[26] Ponce NA, Ku L, Cunningham WE, et al. Language barriers to health care access among Medicare beneficiaries. Inquiry. 2006;43:66–76 16838819 10.5034/inquiryjrnl_43.1.66

[27] Eschbach K, Ostir GV, Patel KV, et al. Neighborhood context and mortality among older Mexican Americans: is there a barrio advantage? Am J Public Health. 2004;94:1807–1812 15451754 10.2105/ajph.94.10.1807 PMC1448538

[28] Rehm J, Greenfield TK, Rogers JD. Average volume of alcohol consumption, patterns of drinking, and all-cause mortality: results from the US National Alcohol Survey. Am J Epidemiol. 2001;153:64–71 11159148 10.1093/aje/153.1.64

[29] Luoto R, Uutela A, Puska P. Occasional smoking increases total and cardiovascular mortality among men. Nicot Tobacco Res. 2000;2:133–139 10.1080/713688127 11072451

[30] Chen Z, Crawford CAG. The role of geographic scale in testing the income inequality hypothesis as an explanation of health disparities. Soc Sci Med. 2012;75:1022–1031 22694992 10.1016/j.socscimed.2012.04.032

[31] Perkins J, Mannix MR, Daniel J, et al. Enforcing language access rights: trends and strategies. Clearinghouse Rev. 2004;38:265

[32] Dowbor T, Zerger S, Pedersen C, et al. Shrinking the language accessibility gap: a mixed methods evaluation of telephone interpretation services in a large, diverse urban health care system. Int J Equity Health. 2015;14:83 26369809 10.1186/s12939-015-0212-9 PMC4570675

[33] El Ansari W, Newbigging K, Roth C, et al. The role of advocacy and interpretation services in the delivery of quality healthcare to diverse minority communities in London, United Kingdom. Health Soc Care Commun. 2009;17:636–646 10.1111/j.1365-2524.2009.00867.x 19486185

[34] Neugroschl J, Sewell MC, Umpierre M, et al. Elderly Latino community members make an educational video: an academic-community collaboration to promote memory evaluations. Int Psychogeriatr. 2019;31:989–995 10.1017/S1041610218001448 PMC6465172 30318026

[35] Masland MC, Lou C, Snowden L. Use of communication technologies to cost-effectively increase the availability of interpretation services in healthcare settings. Telemed eHealth. 2010;16:739–745 10.1089/tmj.2009.0186 PMC2992399 20626299

[36] Torre AC, Bibiloni N, Sommer J, et al. [Spanish translation and transcultural adaptation of a questionnaire on telemedicine usability]. Medicina (B Aires). 2020;80:134–137 32282318

[37] Wosik J, Fudim M, Cameron B, et al. Telehealth transformation: COVID-19 and the rise of virtual care. J Am Med Inform Assoc. 2020;27:957–962 32311034 10.1093/jamia/ocaa067 PMC7188147

[38] Ku L, Flores G. Pay now or pay later: providing interpreter services in health care. Health Affairs. 2005;24:435–444 15757928 10.1377/hlthaff.24.2.435

[39] Karliner LS, Jacobs EA, Chen AH, et al. Do professional interpreters improve clinical care for patients with limited English proficiency? A systematic review of the literature. Health Serv Res. 2007;42:727–754 17362215 10.1111/j.1475-6773.2006.00629.x PMC1955368

[40] Office of Disease Prevention and Health Promotion. (n.d.). Social determinants of health. Healthy People 2030. Rockville, MD: U.S. Department of Health and Human Services

